# Medical Applications of Clostridia and Clostridial Toxins

**DOI:** 10.1155/2012/678963

**Published:** 2012-10-18

**Authors:** Martha L. Hale, Shuowei Cai, S. Ashraf Ahmed

**Affiliations:** ^1^Integrated Toxicology Division, United States Army Medical Research Institute of Infectious Diseases, Fort Detrick, MD 21702, USA; ^2^Biochemistry Department, University of Massachusetts Dartmouth, 285 Old Westport Road, North Dartmouth, MA 02747-2300, USA

The bacterial genus *Clostridium* produces a diverse array of protein toxins, many of which have played an important role in disease and medicine. Although initially known for their ability to do harm, many clostridial toxins have been shown to have therapeutic value for a diverse array of diseases. The articles included in this special issue provide insight into some of that diversity and show that bacterial toxins remain a valuable storehouse for development of new therapeutic measures.

Perhaps the most known toxin therapeutics derived from the clostridial species are neurotoxins secreted by *Clostridium botulinum*. Treatment using BTX is remarkable in that the amount of toxin required is small and the beneficial effects are dramatic, particularly considering the fact that many of the conditions, such as dystonias and muscle spasms, do not respond to other pharmacological treatments. Over the last few decades, BTX has also become cosmetically important and has created significant markets for the drug. Most commercial BTX products are not the pure toxin, but a combination of the toxin and toxin-associated proteins. While these progenitor toxins are safe, a purified form of the toxin would eliminate extraneous proteins and offer a safer drug. Clinical investigations conducted by Y. Matsuka and coworkers (J. Toxicol., 12, 2012) show that BTX purified over a lactose column was an effective treatment for urinary incontinence and prostatic hyperplasia. Their studies provide further data confirming that purified BTX can be used in lieu of progenitor toxins for treatment of many spasmodic conditions. 

Although the focus of most BTX treatment has centered upon using the toxin as a local paralytic to relieve spasm-related illness, new studies show also that BTX aids in the treatment of other difficult-to-treat conditions. D. Intiso (J. Toxicol., 12, 2012) provides an excellent review showing the positive effects of BTX as a supplement in treatment strategies in which the patient requires neurorehabilitative intervention. In treating conditions such as cerebral palsy, stroke, sialorrhea, and other neuromuscular diseases, BTX permits the individual to benefit from physical therapy which is often extremely difficult without some muscle relaxation. New uses of BTX are not limited to rehabilitative intervention, but as suggested by F. j. Lebeda and colleagues (J. Toxicol., 12, 2012), BTX treatment may enhance the processes of wound healing. By performing a systematic search of the literature, the authors developed a qualitative model that shows the possibility of using BTX therapy in wound healing. BTX therapy disrupts muscle spasms. Muscle relaxation around the wound would help to reduce pain and inflammation so that the healing process can proceed more quickly. This work and future studies show that many future treatments for a diverse array of diseases and conditions may benefit from the therapeutic use of BTX. 

While BTX may be the best known, other clostridial toxin-based therapeutics are either currently available or in developmental phases, and three examples are included in this issue. G. Krautz-Peterson et al. and B. Gao and A. McClane utilize clostridial toxin cell binding components as delivery vehicles for therapeutic agents. As with many clostridia toxins, *C. difficile* toxins have a cell binding component and an enzymatic toxic component. These toxins are somewhat unique in that they also contain a protease within the toxin. G. Krautz-Peterson and colleagues show studies that support the ability to link the cell binding region of *C. difficile* toxin B (TcdB) with a therapeutic agent. When the agent is endocytosed, the protease cleaves the drug from the TcdB and is free to act. Additionally, the cell receptor region of TcdB may be replaced with another cell binding receptor, such as the BTX cell receptor region (Hc), thus limiting the cell types to which the TcdB may bind. By combining a BTX inhibitor to the TcdB-Hc, the authors indicate that this drug would provide a BTX inhibitor that is needed to treat botulism. 

Another avenue for utilizing clostridia toxins as therapeutics has been advanced by the investigations of B. Gao and A. McClane (J. Toxicol., 12, 2012) in their development of a therapeutic using *C. perfringens* enterotoxin (CPE). CPE binds to claudin receptors found in tight junctions and cells expressing large amounts of the claudin receptor are particularly susceptible to the cytolytic CPE. Because cancer cells usually express large quantities of claudin on their cell surface, the cells are fairly sensitive to CPE intoxication and CPE provides a natural cytolytic agent for many cancer cells. The primary problem associated with CPE therapy is the fact that the toxin is a protein and, as such, may induce antibodies against CPE which could reduce its effectiveness *in vivo*. B. Gao and A. McClane solve this problem by using the primary receptor domain (C-CPE) to enhance permeability and delivery of chemotherapeutic agents. The truncated size of C-CPE reduces immunogenicity problems while providing a claudin-binding component for the agent. 

The abundance of clostridial toxins with their specific substrates provides a wealth of new pharmacological agents. Combined with the ability to engineer clostridia or its toxins to target specific cells and tissues, the possibilities seem endless. However, the utility of clostridia in medicine does not end with the clostridial proteins. As reviewed by B. Umer et al., the spores of many clostridia species have been developed as cancer therapies. Solid tumors pose a problem to current therapies because the therapeutic cannot be evenly distributed throughout the tumor, partly due to hypoxia and necrotic areas of the tumor. Clostridia spores are strictly anaerobic and will only colonize areas devoid of oxygen and therefore seek out the tumor environment. The spores have been modified to improve their tumor cytolytic capabilities. This review gives an excellent overview of the field and show the possibilities for using clostridia spores in therapy.

As shown by the diverse subjects presented in this special issue, the same characteristics that make Clostridia excellent pathogens have also made the Clostridia an abundant source for development of pharmacological agents. I would like to thank those that contributed to this special issue. Their investigations have given us more insight into the many medical applications of Clostridia and its toxins. Their work provides the foundation for many new and innovative ways in which Clostridia may be harnessed to develop more effective treatments for many diseases and medical conditions.

